# The study of wound healing activity of *Thespesia populnea* L. bark, an approach for accelerating healing through nanoparticles and isolation of main active constituents

**DOI:** 10.1186/s12906-024-04343-2

**Published:** 2024-02-14

**Authors:** Menna B. Abdel Halim, Hanaa H. Eid, Kadriya S. El Deeb, Ghada F. Metwally, Marwa A. Masoud, Omar A. Ahmed-Farid, Hussien M. El Messiry

**Affiliations:** 1 Department of Pharmacognosy, Faculty of Pharmacy, Cairo University, Cairo, Postal code 11562 Egypt; 2 Department of Medicinal plants and Natural Products, Egyptian Drug Authority (EDA; formerly: NODCAR), Giza, Egypt; 3 Department of Pharmacology, Egyptian Drug Authority (EDA; formerly: NODCAR), Giza, Egypt; 4 Department of Physiology, Egyptian Drug Authority (EDA; formerly: NODCAR), Giza, Egypt; 5 Department of Pharmaceutics, Egyptian Drug Authority (EDA; formerly: NODCAR), Giza, Egypt

**Keywords:** Anti-oxidant activity, Chitosan-loaded nanoparticles, Collagen amino acids, *Thespesia populnea* L. bark, Wound healing

## Abstract

The present study provides an evaluation for the wound healing activity of the ethanolic extract of *Thespesia populnea* L. bark (EBE) and its successive fractions in two doses level (1&2%), designed for determining the most bioactive fraction and the suitable dose. Furthermore, development of the most convenient formulation for these bioactive fractions through either their direct incorporation into hydrogel formulations or incorporation of chitosan-loaded nanoparticles with these bioactive fractions into hydrogel formulations. The highest excision wound healing activity was observed in petroleum ether (Pet-B) followed by ethyl acetate (Etac-B) fractions at the high dose (2%). The most suitable formulation designed for the Etac-B fraction was found to be the chitosan-loaded nanoparticles incorporated in the hydrogel formulation, while the conventional hydrogel formulation was observed to be the highly acceptable formulation for Pet-B fraction. Further phytochemical studies of the bioactive fractions led to the isolation of many compounds of different chemical classes viz; beta-sitosterol and lupeol acetate isolated from the Pet-B, in addition to cyanidin and delphinidin from the Etac-B. Our results revealed that EBE and its bioactive fractions (Pet-B & Etac-B) could be considered as strong wound healers through their anti-oxidant and anti-inflammatory activities, in addition to stimulating collagen synthesis.

## Background

The skin is the protective barrier of the organism against physical, chemical and biological impacts from the external environment and any crack (wound) must be rapidly and professionally mended [1]. Wound healing is a complex process that involves four sequential phases; inflammation, proliferation, maturation and ends with scar formation [2]. Reactive oxygen species (ROS) play a pivotal role in the orchestration of the normal wound healing response. It is generated after tissue injury through initiation of acute inflammation or activation of pro-inflammatory cytokine responding to mechanical injury in the acute phase [3]. Clinically, inflammation lasts for 4 days from the time of injury. Both bacteria and its endotoxins present on wounded skin lead to prolonged elevation of pro-inflammatory cytokines and elongate the inflammatory phase, which may result in the formation of chronic wounds. Most wounds heal through the formation of a scar tissue, which is mainly formed of collagen. The remodeling and synthesis of collagen in wound healing process requires energy in the form of adenosine triphosphate (ATP), in addition to, amino acid precursors; glycine, proline, and hydroxyproline, which contribute to 57% of total amino acids in the collagen [4]. Reducing the excessive ROS and inflammatory response of wound in the acute inflammatory stage together with collagen synthesis are key strategies for enhancing the wound healing process [5].

Many of the presented drugs for wound management are not only expensive but also cause problems such as allergy and drug resistance [6]. Thus, plant-based medications have become the subject of up-to-date pharmaceutical formulations for treatment of various ailments, as well as, wound healing as they are reasonably-priced and safe [7].

*Thespesia populnea* (L.) Sol. ex M.P. Correa is a fast growing, medium-sized evergreen tree, with heart-shaped leaves and yellow hibiscus-type cup-shaped flowers having maroon color at the center. It is commonly called Portia tree, Pacific rosewood and Indian tulipe tree [8]. Rao et al., (2018) [9] reviewed the traditional use of fruits, flowers and leaves topically for treatment of scabies, psoriasis, eczema and herpetic diseases, earlier studies concerning different extracts of heart wood and leaves of *Thespesia populnea* L. revealed their activity to restore GSH level in alcohol-induced oxidative stress in rats [10], and reduction of levels of MDA and MPO [11]. Furthermore, lipid peroxidation was highly reduced after treatment with aqueous and methanolic extracts of the bark [12], as well as after use of ethanolic extract of the leaves [10]. Hot poultice of the leaves is used on inflamed and swollen joints, while bark was traditionally used to treat dysentery, hemorrhoids and skin diseases. In addition, Rao et al., (2018) [9] reviewed the bark to possess anti-inflammatory, anti-microbial, anti-oxidant and wound healing activities. The complexity of the wound healing process necessitates the use of both in-vitro and in-vivo models, however, in-vitro models using cell cultures are limited in their ability to evaluate dynamic wound healing processes viz. hemostasis and angiogenesis, despite providing complete control over environmental elements such as temperature or fake wound size, they are incapable of reproducing biological circumstances [13]. Previous studies revealed that *Thespesia populnea* bark ethanolic extract exerted many biological activities that were performed by means of in-vivo studies viz. the antidiabetic potential of the extract in streptozotocin-induced diabetic rats through reducing blood glucose level and improving insulin sensitivity via anti-oxidant action [14]. The anti-inflammatory and analgesic properties of the bark extract was found to be exerted through reducing pain responses using the model of acetic acid-induced writhing response and formalin-induced paw licking time in the early and late phases [15]. The ethanolic bark extract also proved to possess a hepatoprotective effect against carbon tetrachloride (CCl4)-induced liver injury in rats, indicating its potential effect for liver protection and regeneration [16]. The antioxidant properties of the bark ethanolic extract was demonstrated by scavenging free radicals and reducing oxidative stress [12]. The findings from previous studies were in accordance with the aim of our present work concerning the use of ethanolic extract of the bark of *Thespesia populnea* to study its wound healing activity that may be exerted by its anti-oxidant and anti-inflammatory activities as proved previously. Furthermore, previous wound healing studies were performed on *Thespesia populnea* using excision and incision wound model on animals, but by using different extracts from different organs of the plant as: aqueous extract of the fruit [17], alcoholic, aqueous and petroleum ether extracts of leaves [18] and finally bark powder [19], that all proved the strong wound healing activity of the plant. As a result, in-vitro models were excluded from this study.

A combination of many wound healing models would improve the reliability and validity of the data and also provide a better knowledge of the mechanisms involved in tissue repair, however, excisional wounds are one of the most commonly used models that represent acute clinical wounds, where no sutures are required. This model can study many parameters viz. hemorrhage, inflammation, granulation tissue development, in addition to, rate of wound contraction can be tracked easily with the ease of application of topical drugs on the wound area. On the contrary, the incision wound model is more useful in the study of surgical incision materials through tensile strength evaluation and measuring other parameters. In our study, the authors selected the excision model as they were interested in studying the acute wound model regarding rate of wound contraction, histopathological study and measuring biochemical parameters covering the aspects of anti-oxidant, anti-inflammatory activities and collagen amino acids formation in order to fully investigate all the features of wound healing activity in one model [20].

Thus, the aim of this study was to evaluate the wound healing potential of *Thespesia populnea* L. ethanolic bark extract and its successive fractions in two doses level to determine the most bioactive fractions together with the most convenient dose, as well as, isolating the bioactive metabolites. In addition, the hydrogel formulations containing directly these bioactive fractions and the bioactive fractions encapsulated in chitosan nanoparticles were evaluated for their wound healing activity.

## Material and methods

### Plant material

The bark of *Thespesia populnea* (L.) Sol. ex M.P. Correa was collected from Al Qanatir Al Khayriyyah nurseries, Al-Qalyubiyya Governorate, Egypt, during the flowering stage in September 2020. The plant was authenticated by Mrs. Therese Labib, Botanical Specialist and Consultant at Orman and Qubba Botanicals Gardens. The identity of the plant material was confirmed by Prof. Dr. Reem Samir Hamdy, Professor of Taxonomy and Flora, Botany & Microbiology department, Faculty of Science, Cairo University (Specimen number: 9.11.2022 in the herbarium of Faculty of Pharmacy, Cairo University).

### Preparation of ethanolic extract of bark and its successive fractions

Two kilograms of the air-dried powdered bark was extracted by 95% ethanolic solution by cold maceration, the extract was concentrated under reduced pressure and dried to afford 615 g of the ethanolic bark extract (EBE), (30.75% w/w on dry weight basis). The extract was subjected to preliminary phytochemical screening tests adopting procedures according to Dubale et al.***,*** 2023 [21]. The reddish brown EBE was suspended in water and partitioned successively with solvents of increasing polarity to yield 170.52 (27.73%), 8.83 (1.44%), 109.25 (17.76%) and 153.75 g (25%) of petroleum ether (Pet-B), methylene chloride (Met-B), ethyl acetate (Etac-B) and butanol (But-B) fractions, respectively. For chemical investigation and preparation of formulations, EBE and its successive fractions were stored at − 20 °C until use. For biological evaluation, they were separately incorporated in hydrogel formulations at concentration 1 & 2% (w/w) to determine the bioactive fractions and kept in the refrigerator.

### Drugs and chemicals

Butanol, Ethanol, Ethyl acetate, Formalin, Methylene chloride and Petroleum ether (ADWIC, Cairo, Egypt), Saline (Sigma-Aldrich, Cairo, Egypt), Thiopental sodium (Sigma-Aldrich, Cairo, Egypt).

 Kits: Malondialdehyde, Nitric Oxide assay, Reduced Glutathione (Biodiagnostic, Giza, Egypt).

### Preparation of hydrogel formulations

#### Hydrogel formulations for screening of biological activity

Hydrogel formulations containing EBE (1 and 2%) and its successive fractions were prepared as follows: firstly, by sprinkling weighed amount of carbopol 934 (0.5%) into a beaker 100 ml containing purified water at 40 °C using a mixer, at 1200 rpm, for 90 min to produce the hydrogel base. Secondly, EBE and its fractions were dispersed, separately, in Polyethylene glycol 400 and added to the hydrogel base and mixed well. The pH was then adjusted to pH 6 using triethanolamine and stirred slowly until a clear and transparent hydrogel was obtained [22].

#### Preparation of chitosan nanoparticles loaded with bioactive fractions

The most important characteristics of nanoparticles are their small particle size together with their ability to control drug release. In wound healing, nanoparticles are either used as a delivery vehicle or as a bioactive component [23], where the drug itself may be formulated at a nanoscale such that it can function as its own carrier [24]. In this study the nanoparticles were prepared to demonstrate the effect of decreasing the particle size on the efficacy of the bioactive fractions related to their wound healing activity, together with the selection of chitosan as vehicle to load the bioactive fraction and its effect on the improvement of the wound healing activity, as it could play a role being iteslf a wound healer [25].

The ionic gelation method [26] was used for the preparation of chitosan-loaded nanoparticles from both selected bioactive fractions; Pet-B and Etac-B to obtain Pet-B-CLN and Etac-B-CLN, respectively. Chitosan (2% w/v) was dissolved in 1% acetic acid and cross linking agent sodium tripolyphosphate (2%, w/v) was added dropwise in the solution while stirring, at room temperature. Opalescent color solution was observed then stirring was continued for 60 min. Loading of Pet-B & Etac-B fractions to chitosan nanoparticles was performed by dissolving 2 g of each fraction separately in 100 ml ethanol, then the formed solution was added dropwise to the chitosan solution while stirring for 2 hours, followed by centrifugation at 5000 rpm for 10 minutes. Pellets were re-suspended in phosphate buffer, nanoparticles were collected after centrifugation at 10000 rpm for 10 minutes and then washed with distilled water. The obtained Pet-B-CLN was composed of brownish green pellets (2.5 g) while Etac-B-CLNs formed of reddish brown pellets (2.2 g), the colours of the obtained nanoparticles are in accordance with that of the fraction from which they were prepared.

#### Evaluation of the prepared nanoparticles

Pet-B-CLN and Etac-B-CLN were evaluated for their particle size (PI), zeta potential (ZP) and polydispersity index (PDI) using a laser light scattering-based particle size analyzer (Zetasizer Nano ZS, Malvern, UK), to ascertain that the produced nanoparticles possess the characteristics that help in exerting the required biological activity. Analysis was performed in triplicate, and average values with standard deviation are recorded (Table 2). Encapsulation efficiency (EE) was determined for the prepared nanoparticles and expressed as the marker percentage (main active constituent; β-sitosterol in Pet-B and total flavonoids as rutin in Etac-B fractions) [27]. EE and loading capacity (LC) of plant fractions were determined according to Hassan et al. (2019) [28] by the following equations:

$$\text{EE}\;\left(\%\right)=\frac{mass\;of\;initially\;added\;drug-mass\;of\;free\;drug}{mass\;of\;initially\;added\;drug}\times100$$

$$\begin{array}{c}\text{LC}\;\left(\%\right)=\frac{mass\;of\;initially\;added\;drug\;-\;mass\;of\;free\;drug}{Nanoparticles\;weight}\times100\\\end{array}$$

### Preparation of formulations F1-F4

Hydrogel formulations (F1-F4) were prepared from the selected bioactive fractions, as previously mentioned [22] and presented in (Table 1), the prepared hydrogel base was similar to that of the reference drug used (Panthenol gel).

**Table 1:** The composition of the prepared hydrogel formulations of the bioactive fractions

Ingredients (g)	Prepared hydrogel formulations			
	F1	F2	F3	F4
Carbopol 934	0.5	0.5	0.5	0.5
Ethanol (ml)	20	20	20	20
Polyethylene glycol 400	20	20	20	20
Pet-B	2	–	–	–
Etac-B	–	–	2	–
Pet-B-CLN	–	2	–	–
Etac-B-CLN	–	–	–	2
Triethanolamine (ml)	QS	QS	QS	QS
Water (quantity sufficient- ml)	100	100	100	100

*QS* Quantity sufficient for pH adjustment

*Pet-B* petroleum ether fraction: *Etac-B* ethyl acetate fraction, *Pet-B-CLN* petroleum ether fraction encapsulated in chitosan nanoparticles, *Etac-B-CLN* Ethyl acetate fraction encapsulated in chitosan nanoparticles

#### Evaluation of the prepared hydrogel formulations (F1 & F4)

Physicochemical evaluations of the hydrogel formulations (F1 & F4) that showed the highest activity and were selected after evaluation of their biological activity including physical properties, spreadability, rheological properties and percentage of active constituents.

##### Physical properties

F1 & F4 were inspected visually for their colour, odour, homogeneity and transparency.

##### Spreadability

The spreadability of F1 & F4 were separately determined after 2 days from preparation, by calculating the difference in the diameter in (mm) before and after the application of glass plates [29] (Table 3).

##### Rheological properties

**Fig. 1:**
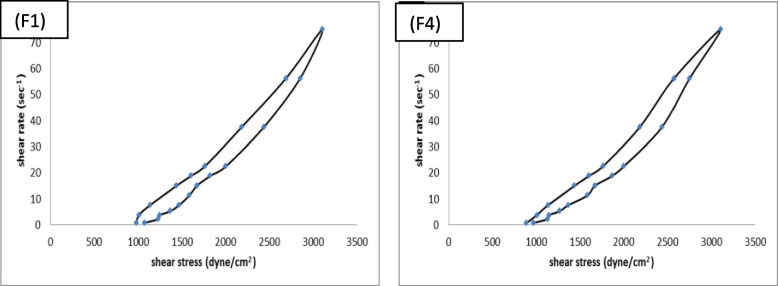
Rheograms of the prepared hydrogel formulations: F1 & F4. F1: hydrogel containing petroleum ether fraction, F4: hydrogel containing chitosan loaded ethyl acetate nanoparticles

The rheological properties of F1 & F4 were determined at 25 °C using cone and plate viscometer (Brookfield DV-III Ultra programmable Rheometer, UK), spindle 40 connected to water bath (Polyscience, USA) adjusted to 25 °C. One gram of each hydrogel formulation was tested to determine the flow behavior and the viscosity at different rates of shear. The shear stress in dyne/cm^2^, shear rate in sec^−1^ and viscosity in centipoises (cp) were determined (Table 3 & Fig. 1).

### In-vivo pharmacological study

#### Animals

Adult male Albino rats (150 ± 20 g) were obtained from the breeding colony maintained at the animal house of the Egyptian Drug Authority (EDA, Cairo, Egypt). Animals were housed for at least 1 week in the laboratory room prior to testing under standard housing conditions (room temperature 24–27 °C) with alternating 12 hour light and dark cycles and were allowed free access to food (standard pellet diet) and water*,* unless otherwise specified. Animals were treated in strict compliance with the international laws on the use and care of experimental animals. The study was approved by the Ethics Committee of Faculty of Pharmacy, Cairo University [MP (2257)]. Euthanasia of animals was performed by an overdose of sodium pentobarbital (800 mg/kg) [30].

#### Acute dermal toxicity

The OECD guidelines [**N° 402, 2017]** [31], were followed for the study. A total of six rats (3 female and 3 male) were used. The limit test dose of 2000 mg/kg formulation was applied uniformly over the shaved area. After the exposure period, the clinical signs and mortality were monitored at 30 minutes, 2, 4, 6 and 24 hours and once daily for 14 days.

#### Evaluation of wound healing activity

Evaluation of wound healing activity of EBE and its fractions were performed on two stages (I & II).

##### Stage I: assessment of the EBE and its fractions for screening of wound healing activity

A total of 130 albino rats were randomly divided into thirteen groups, (10/each) [32]. Group (1): non-wounded rats remain all the experiment without treatment (normal control), Group (2): wounded non-treated without receiving any treatment (positive control), Group (3): wounded rats, treated with hydrogel base (vehicle), Group (4): wounded rats, treated with EBE at dose 1%, Groups (5–8): wounded rats, treated with successive fractions; (Pet-B, Met-B, Etac-B and But-B at dose 1% in hydrogel base, respectively, Group (9): wounded rats, treated with EBE at dose 2%, Groups (10–13): wounded rats, treated with successive fractions at dose 2% in hydrogel base, respectively. This step is considered as a screening one to determine the wound healing activity of the studied fractions at two doses level (1 & 2% in hydrogel base) in order to identify the most bioactive fraction and the most appropriate dose, compared to normal, positive (untreated) and gel base (vehicle) groups.

##### Stage II: assessment of the prepared hydrogel formulations (F1-F4) of the bioactive fractions at dose 2%

A total of 80 albino rats were randomly divided into eight groups (G1-G8), (10/group), Group (1): non-wounded rats remain all the experiment without treatment (normal control), Group (2): wounded non-treated without receiving any treatment (positive control), Groups (3–8): wounded rats treated with vehicle (hydrogel base), Panthenol gel (reference drug) and formulations (F1-F4), respectively.

The wound healing model in both stages I & II was performed according to Shahein et al.***,*** (2017) [33]. The rats were anaesthetized with 50 mg/kg thiopental sodium via intraperitoneal injection. The dorsal surface of rats was shaved, cleaned with 70% ethanol. Circular excision wounds were made by cutting out predetermined dorsal area (≈22 mm diameter) of skin from the shaved area using toothed forceps and pointed scissors. The entire wound was covered with sterile gauze attached to the ends with tape to prevent the animal from withdrawing or ingesting the investigated sample for 24 h. One gram of each of the prepared formulations (equivalent to 10 & 20 mg extract / fraction), hydrogel base (vehicle) and standard drug (Panthenol gel) were topically applied once daily after skin excision for 14 successive days. Wound contraction area was measured on days 2, 5, 7, 11 and 14 in each group, according to Esimone et al., (2006) [34]. The reduction of wound area, at every other day, for each wounded rat for 14 days was measured using transparency paper and a marker, the wound percentage of contraction was calculated as follows:

$$\mathrm{Wound}\;\mathrm{contraction}\;\left(\%\right)\;=\:\left\{\left(\mathrm{wound}\;\mathrm{area}\;\mathrm{on}\;\mathrm{day}\;0\;{-}\;\mathrm{wound}\;\mathrm{area}\;\mathrm{on}\;\mathrm{day}\;\mathrm n\right)/\mathrm{wound}\;\mathrm{area}\;\mathrm{on}\;\mathrm{day}\;0\right\}\:\times\:100$$

Where day 0: the day of wounding before treatment, day n: day 2, 5, 7, 11 or 14 after treatment.

##### Assessment of the biochemical parameters

Half of the animals in each group were euthanised on day 7 and the rest of the animals were euthanised on the 14th day and tissue samples were collected and washed with ice-cold saline. Portions of the skin tissue were homogenized in ice-cold saline producing 10% homogenate. The homogenates were centrifuged at 4000 rpm at 4 °C for 15 min then the supernatants were separated and used for biochemical analyses.

The determination of oxidative stress markers as reduced glutathione (GSH) [35], malondialdehyde (MDA) [36] and nitric oxide (NO) [37] was undergone, while the inflammatory marker myeloperoxidase activity (MPO) was determined according to method of Bradley et al. (1982) [38] and NF-Kβ according to ELISA kit procedure. Moreover, Glycine, Proline, Hydroxyproline were determined by HPLC/UV detector [39].

##### Histopathological study

Autopsy samples were taken from the skin of rats in different groups, at days 7 and 14 and fixed in 10% formaldehyde solution for 24 hours according to Bancroft et al., (1996) [40].

### Statistical analysis

Data are presented as mean ± SEM (standard error of the mean). Statistical analysis was carried out by one-way analysis of variance (ANOVA) followed by Tukey-Kramer as a post hoc test. Analysis were performed using a GraphPad prism computer program (GraphPad software Inc. V5, San Diego, CA, USA). Statistical significance was accepted at *P* < 0.05 level.

### Phytochemical study of the bioactive fractions

Phytochemical investigation was performed on the most bioactive fractions viz. Pet-B and Etac-B in order to isolate the main active constituents.

#### Chromatographic isolation and identification of the constituents from pet-B

Pet-B (100 g) was chromatographed on column of alumina (WN-3, 70–290 mesh, Sigma Aldrich) using petroleum ether, methylene chloride, ethyl acetate and finally methanol (gradual elution was adopted, increase by 5%). Fractions (50 ml) were collected to yield 136 subfractions, monitored by TLC using different solvent systems (the best resolution was displayed by toluene: ethyl acetate; 7:3 v/v) and were pooled to give 28 major fractions (MF). Nine promising fractions (MF: 10, 12, 14, 16, 19, 23, 25, 26 & 28) were subjected to different chromatographic techniques that led to isolation of nine compounds (P1 – P9), where one compound was isolated from each fraction.

#### Chromatographic isolation and identification of the constituents from Etac-B

Etac-B (100 g) was chromatographed on a VLC column (5 X 65 cm) packed with 100 g silica gel G60 (60–120 mesh, Merck, Darmstadt, Germany) and elution was performed starting from methylene chloride 100% then gradually increased by 2.5% using ethyl acetate, methanol, water until reaching methanol: water; 50: 50 (v/v). Fractions (50 ml) were collected to yield 53 subfractions, then monitored by TLC (the best solvent system: n-butanol; acetic acid: water; 4: 1: 5 (v/v/v) upper phase). Similar fractions were pooled together to give 15 major collective fractions (CF). Six collective fractions (CF: 3, 8, 12, 13, 14 & 15) were promising and were re-chromatographed on Sephadex LH-20 (Mitsubishi Kagaku, Tokyo, Japan) (50 g, 2 X 25 cm), gradient elution was carried out (by increasing polarity by 5%) using methylene chloride, ethyl acetate, methanol and water up to 50% methanolic solution, that led to isolation of fifteen compounds (E1 – E15), where three compounds were isolated from each of CF: 3, 8 & 12, while CF: 13, 14 and 15 afforded two compounds each; (E10-E15), respectively.

#### Structural elucidation of the isolated compounds

The isolated compounds (P1-P-9) & (E1-E15) were identified based on their physical properties and spectral data (UV, ^1^H-NMR &^13^C-NMR), compared with those in the published literature. UV spectrophotometery was measured using a Unicam (Thermo, UK) spectrophotometer and ^1^H-NMR &^13^C-NMR was determined using Bruker magnetic resonance (400 MHZ, USA).

#### Quantitative estimation of β-sitosterol in pet-B fraction (marker)

β-sitosterol was chosen as a marker for the determination of Pet-B as it is isolated in considerable amount from Pet-B together with its various biological activities viz. wound healing, anti-oxidant, anti-inflammatory or anti-microbial activities. It was determined by HPTLC (CAMAG) in Pet-B according to Mallick and Dighe (2014) [41].

#### Quantitative estimation of flavonoids in Etac-B fraction (marker)

Total flavonoids calculated as rutin were chosen as a marker for Etac-B due to the richness of Etac-B with polyphenolic compounds especially flavonoids (rutin was proved to be present in high amount in Etac-B) and their known activity as potent anti-oxidant and free radical scavenging activities. Total flavonoids as rutin were estimated in the Etac-B by the aluminium chloride colorimeteric assay [42] using UV spectrophotometer (Unicam, Thermo, UK). Results are compiled in (Table 2). Results are presented in (Table 2) & expressed as EE and loading capacity results.

**Table 2:** Evaluation of the prepared petroleum ether & ethyl acetate fractions loaded in chitosan nanoparticles

Nanoparticles	Particle size (nm)	Zeta potential (mv)	Polydispersity index	Encapsulation Efficiency	Loading capacity
**Pet-B-CLN**	41.69 ± 2.06	33.3	0.69	82.5%	34.04%
**Etac-B-CLN**	96.66 ± 3.67	− 22.6	0.95	92.5%	42.05%

*Pet-B-CLN* petroleum ether fraction encapsulated in chitosan nanoparticles, *Etac-B-CLN* Ethyl acetate fraction encapsulated in chitosan nanoparticles

## Results

### Preliminary phytochemical screening

Phytochemical screening of the EBE revealed the presence of carbohydrates, tannins, flavonoids, saponins, sterols, terpenes, alkaloids and cardiac glycosides.

### Characterization of hydrogel formulations

#### Evaluation of the prepared nanoparticles

The obtained results (Table 2) showed that Pet-B-CLN showed better results than that obtained from Etac-B-CLN concerning particle size (41.69 nm) and polydispersity index (0.69) that indicate a uniform distribution of particles, while that of Etac-B-CLN showed particle size (96.66 nm) and polydispersity index (0.95). The polydispersity index is a measure of the heterogeneity of a sample based on size. It represents size distribution in a sample or agglomeration or aggregation of the sample during isolation or analysis, the higher of its value indicates a broad size distribution of particles, this may have effect on the nanoparticle release from dosage form and its absorption or penetration. Zeta potential of PET-B-CLN (33.3 mv) is higher than that of Etac-B-CLN (− 22.6 mv). Furthermore, Etac-B-CLN (Table 2) showed higher results concerning encapsulation efficiency (92.5%) than that of Pet-B-CLN (85.15%) and loading capacity (42.05%), while Pet-B-CLN showed (34.04%), throwing lights that flavonoids that are present in Etac-B were highly loaded on chitosan nanoparticles than the sterols of Pet-B.

### Pharmaceutical evaluation of the selected most convenient hydrogel formulations (F1 &F4)

#### Physical properties

The prepared hydrogel formulations were brown in colour, homogenous, with characteristic odour. The visual inspection shows no noticeable difference in the hydrogel formulations prepared directly from plant fractions or that were loaded on chitosan nanoparticles.

##### Spreadability

Spreadability is an index of the release of the correct amount of drug to the target tissue and the ease of application of the hydrogel. The spreading diameter of hydrogel formulations (Table 3) ranges from 15 to 17 mm.

**Table 3:** Physical properties of hydrogel formulations (F1 & F4)

Formulation	Viscosity		Spreading diameter (mm)
	η_**min**_^**a**^ (cp.)	η_**max**_^**b**^ (cp.)	
**F1**	202,415	21,053	17
**F4**	193,729	20,983	15

*F*1 hydrogel containing petroleum ether fraction, *F*4 hydrogel containing chitosan loaded ethyl acetate nanoparticles

^a^Viscosity at low rate of shear, ^b^Viscosity at high rate of shear

##### Rheological properties

Table 3 showed the viscosity of each formulation at minimum rate of shear (η_min_) and at maximum rate of shear (η_max_). It is clear that the (η_max_) was less than (η_min_). This finding proved the structural breakdown of hydrogel formulations during shear. The (η_max_, η_min_) and spreadability did not show noticeable difference between the prepared formulations using the same hydrogel base.

It was also found (Fig. 1) that the prepared hydrogel formulations showed plastic flow (curve doesn’t start from the origin).

This finding proved the structural breakdown of gel formulations during shear. The curves that graphically represented in Fig. 1 showed that; shear stress vs shear rate showed significant hysteresis loops, while «ascending» curve, which describes the gel destruction, differ from the «descending» curve, which indicates the resumption of the gel, this is explained by preservation of residual deformation after a strong attenuation of the structure under the influence of earlier applied stress. Hysteresis loop curves indicates that the gel possess thixotropic properties. The presence of thixotropic properties in gel characterizes satisfactory spreadability on the skin, active constituent release from gel base to skin and the ability of extrusion from the tube [43].

### Acute dermal toxicity

The acute dermal toxicity test of EBE and its fractions showed no signs of toxicity or mortalities at doses up to 2000 mg/kg. Therefore, hydrogel formulations of 1000 mg/100 g & 2000 mg/100 g were prepared and used for the in-vivo pharmacological evaluation [44].

### In-vivo assessment of wound healing activity

#### Assessment of ethanolic extract and its fractions

The obtained data (Table 4) showed the effect of EBE extract and its fractions of studied plant referenced to vehicle (hydrogel base) on excision wound model. Presented data revealed recovery procrastination for positive control and vehicle groups till 14th day after induction. In contrast, the groups treated with EBE, Pet-B, and Etac-B, at dose 2%, showed accelerated recovery within 14 days of treatment while lower dose showed less effect. Meanwhile, Met-B, and But-B treated groups at both doses showed mild amelioration of excision wound nearly to vehicle group. It was observed that the high dose (2%) showed much better results than that of low dose (1%) in all groups.

**Table 4:** Effect of the ethanolic bark extract (EBE) and its successive fractions (1 & 2%) of *Thespesia populnea* (L.) on wound area contraction in circular excision wound model (*n* = 10)

Groups		Dose (%)	Wound contraction (%)				
			Day 2	Day 5	Day 7	Day 11	Day 14
**G(1)**	**Normal Control**	–	–	–	–	–	–
**G(2)**	**Positive control (untreated)**	–	7.31^C^	14.8 ^g^	48.6^C^	62.1 ^C^	71.92^d^
**G(3)**	**Vehicle hydrogel base**	–	9.13 ^b^	27.54 ^d^	36.60^d^	69.24 ^b^	74.8^C^
**G(4)**	**EBE**	1	8.56 ^bC^	21.60 ^f^	54.20^c^	82.98^**a**^	90.10^**a**^
**G(5)**	**Pet-B**	1	7.22 ^c^	30.13 ^d^	57.96 ^c^	83.73 ^a^	**94.55** ^b^
**G(6)**	**Met-B**	1	9.11^b^	31.48 ^d^	53.80 ^c^	81.58 ^b^	87.18 ^b^
**G(7)**	**Etac-B**	1	8.54 ^bc^	24.70 ^de^	60.40 ^b^	82.14 ^b^	91.43 ^a^
**G(8)**	**But-B**	1	9.76^b^	16.10 ^g^	37.71 ^d^	53.97 ^d^	72.94 ^d^
**G(9)**	**EBE**	**2**	**15.40**^**a**^	**25.94** ^**de**^	**65.60**^**a**^	**85.90**^**a**^	**93.30**^**b**^
**G(10)**	**Pet-B**	**2**	**9.58** ^**b**^	**35.42**^**c**^	**62.05** ^**ab**^	**83.70**^**a**^	**95.10** ^b^
**G(11)**	**Met-B**	2	9.65 ^b^	39.30 ^**b**^	61.5 ^b^	79.23^**a**^	87.45^**a**^
**G(12)**	**Etac-B**	**2**	**9.78** ^**b**^	**44.30**^**a**^	**63.77**^**ab**^	**84.87** ^**a**^	**93.10**^**b**^
**G(13)**	**But-B**	2	10.95 ^b^	24.73^e^	39.7 ^d^	54.84 ^d^	73.24^C^

Values are expressed as mean, values with different superscript letters (a-g) in the same column at the same interval differ significantly (*P* < 0.05), Normal control: healthy group, Positive control: untreated group, Vehicle: hydrogel base

*Pet-B* petroleum ether, *Met-B* methylene chloride, *Etac-B* ethyl acetate, *But-B* butanol fractions

#### Assessment of the prepared hydrogel formulations

From the previous results, it was concluded that Pet-B and Etac-B fractions at dose 2% showed the highest wound healing activity, so hydrogel formulations containing these fractions or their chitosan-loaded nanoparticles were prepared and their wound healing activities were assessed.

##### Wound contraction

**Fig. 2:**
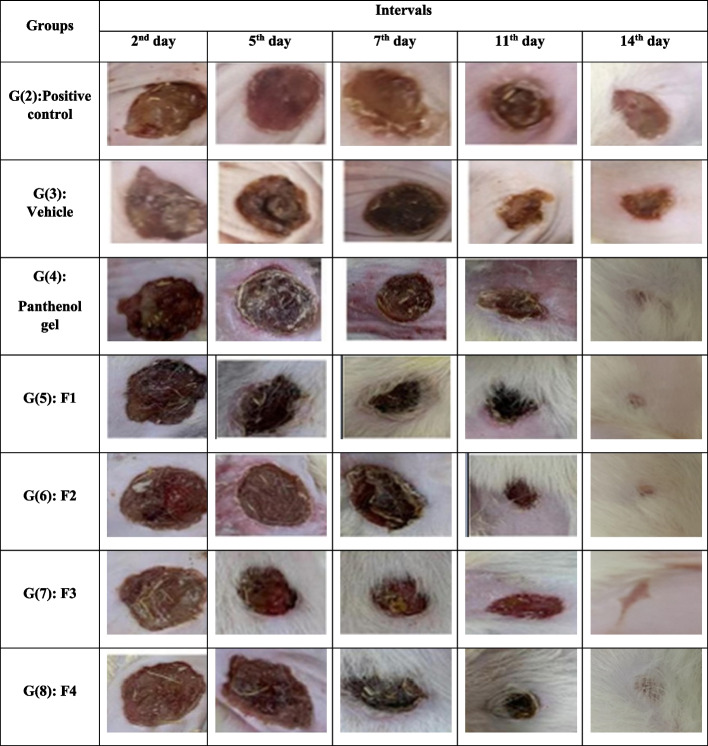
Photos showing wound contraction activity of the F1-F4 against Panthenol gel, positive control and vehicle (gel base) groups in circular excision wound model on days 2, 5, 7, 11 and 14. F1: hydrogel containing petroleum ether fraction, F2: hydrogel containing chitosan loaded petroleum ether nanoparticles, F3: hydrogel containing ethyl acetate fraction, F4: hydrogel containing chitosan loaded ethyl acetate nanoparticles

The obtained data (Fig. 2) showed that contraction of wounds didn’t show any differences between groups at 1st interval (Day2). The groups treated with F1 and F4 showed higher effect on day 7 compared to other groups, meanwhile, F4 showed high wound healing action as it accelerated wound contraction through the 14 days. F1 showed better significant effect on wound contraction than that exerted by F2 through the days of treatment, except on day 14, F1 showed nearly the same effect exerted by F2. Meanwhile, F4 showed better results than that obtained by F3 which is significant on almost all days of treatment. In addition, F4 and reference treatment (Panthenol gel) showed similar amelioration at nearly most intervals and the F1 group accede to them at 4th, and 5th intervals (11 & 14th days, respectively).

##### In-vivo assessment of biochemical parameters

The obtained data (Table 5) showed marked amelioration for F1 and F4-treated groups which was resembled in the decrease of the oxidative stress and inflammatory markers and enhancing the collagen building with precursor amino acids (Glycine, Proline and hydroxyproline) and increasing GSH on both days 7 and 14. F1 and F4 showed high activity in lowering MDA, NO, NF-Kβ, MPO, and increasing GSH, Gly, Pro, and OH-Pro, followed by F2. In the same manner, reference drug (panthenol gel) and other treated groups showed accelerated recovery but still lower than that of F1 and F4.

**Table 5:** Effect of the hydrogel formulations (F1-F4) on serum levels of oxidative stress (GSH, MDA & NO), inflammatory (NF-Kβ & MPO) and amino acids of collagen markers in circular excision wound model (*n* = 10)

Intervals	Groups		Biochemical Parameters							
			Oxidative stress			Inflammatory		Amino acids of collagen		
			GSH (μmol/g tissue)	MDA (nmol/ g tissue)	NO (μmol/g tissue)	NF-Kβ (pg/ g tissue)	MPO (U/mg protein)	Gly (μg/ g tissue)	Pro (μg/ g tissue)	OH-Pro (μg/ g tissue)
**7th day**	**G (1)**	**Normal control**	2.45 ± 0.27^c^	3.22 ± 0.47^a^	35.79 ± 2.23^a^	607 ± 30.2^a^	0.48 ± 0.057^b^	60.75 ± 4.78^c^	20.47 ± 2.48^b^	9.90 ± 0.61^c^
	**G (2)**	**Positive control**	0.82 ± 0.14^e^	7.68 ± 0.57^c^	133.8 ± 15.5^c^	1591 ± 84.2^c^	5.74 ± 0.828^d^	19.63 ± 1.50^d^	8.08 ± 0.90^c^	3.50 ± 0.36^d^
	**G (3)**	**Vehicle**	1.01 ± 0.11^d^	8.11 ± 0.91^a^	112.2 ± 12.9^b^	1639 ± 86^a^	4.1 ± 0.391^b^	24.28 ± 2.19^ef^	8.29 ± 0.86^d^	3.95 ± 0.44d^e^
	**G (4)**	**Panthenol gel**	2.82 ± 0.07 ^c^	2.84 ± 0.06 ^a^	28.38 ± 0.44^a^	612 ± 33.1^a^	0.53 ± 0.01^a^	62.55 ± 1.84^c^	23.5 ± 0.63^b^	10.94 ± 0.27^a^
	**G (5)**	**F1**	**2.97 ± 0.08**^**c**^	**2.57 ± 0.05** ^**ab**^	**24.57 ± 0.72**^**b**^	**530 ± 24** ^**b**^	**0.38 ± 0.01**^**b**^	**66.93 ± 2.38**^**b**^	**25.47 ± 0.37**^**a**^	**11.21 ± 0.24**^**a**^
	**G (6)**	**F2**	2.84 ± 0.09 ^a^	2.74 ± 0.07 ^b^	26.35 ± 0.5 ^b^	535 ± 26.2 ^b^	0.46 ± 0.01^c^	65.21 ± 2.06^a^	25.17 ± 0.63^a^	11 ± 0.21^a^
	**G (7)**	**F3**	2.56 ± 0.08 ^d^	2.82 ± 0.07 ^a^	31.79 ± 0.69^a^	576 ± 24.4 ^a^	0.47 ± 0.01^b^	61.05 ± 1.95^c^	22.55 ± 0.58^b^	10.15 ± 0.25^a^
	**G (8)**	**F4**	**3.25 ± 0.08** ^**b**^	**2.43 ± 0.07** ^**b**^	**24.54 ± 0.55**^**b**^	**514 ± 24.3**^**ab**^	**0.3 ± 0.01**^**c**^	**70.28 ± 1.9**^**b**^	**26.99 ± 0.68**^**a**^	**11.28 ± 0.36**^**a**^
**14th day**	**G (1)**	**Normal control**	2.57 ± 0.24^c^	2.67 ± 0.32^a^	28.65 ± 2.54^a^	578 ± 27.3^a^	0.39 ± 0.055^a^	67.28 ± 6.59^d^	22.43 ± 3.12^d^	10.7 ± 0.70^b^
	**G (2)**	**Positive control**	0.91 ± 0.20^d^	6.39 ± 0.51^c^	103.6 ± 11.9^c^	1363 ± 74.4^c^	4.49 ± 0.879^c^	21.18 ± 1.75^e^	8.77 ± 1.51^e^	3.82 ± 0.48^c^
	**G (3)**	**Vehicle**	1.14 ± 0.18^d^	6.74 ± 0.83^a^	88.09 ± 6.49^b^	1335 ± 72.2^a^	3.23 ± 0.357^b^	26.65 ± 2.73^de^	9.01 ± 0.93^d^	4.49 ± 0.38d^e^
	**G (4)**	**Panthenol gel**	3.37 ± 0.12^b^	2.47 ± 0.04^a^	27.11 ± 0.68^a^	558 ± 24.9^a^	0.43 ± 0.01^a^	73.87 ± 1.86^c^	27.15 ± 0.8^c^	13.31 ± 0.34^a^
	**G (5)**	**F1**	**3.76 ± 0.08**^**b**^	**2.18 ± 0.08**^**b**^	**22.63 ± 0.63**^**b**^	**488 ± 24.4**^**b**^	**0.33 ± 0.01**^**a**^	**79.7 ± 2.09**^**b**^	**31.63 ± 0.72**^**ab**^	**13.49 ± .16**^**ab**^
	**G (6)**	**F2**	3.4 ± 0.09^a^	2.28 ± 0.03^b^	22.98 ± 0.57^b^	491 ± 19.9^b^	0.4 ± 0.01^b^	76.22 ± 2.07^a^	31.02 ± .51^ab^	12.54 ± 0.26^a^
	**G (7)**	**F3**	2.87 ± 0.04^c^	2.52 ± 0.03^a^	27.23 ± 0.54^a^	518 ± 21.7^a^	0.41 ± 0.01^a^	76.17 ± 2.75^b^	29.54 ± .64^bc^	11.55 ± 0.32^b^
	**G (8)**	**F4**	**4.17 ± 0.1**^**b**^	**2.15 ± 0.05**^**b**^	**21.36 ± 0.56**^**b**^	**424 ± 26.8**^**b**^	**0.29 ± 0**^**b**^	**81.72 ± 1.55**^**a**^	**33.54 ± 0.3**^**a**^	**13.99 ± 0.31**^**a**^

Values are expressed as mean ± S.E.M (standard error of means); values having different superscript letters (a-f) in the same column at the same interval differ significantly (*P* < 0.05). Normal control: Healthy group, Positive control: untreated group, Vehicle: gel base

*GSH* glutathione, *MDA* malondialdehyde, *NO* nitric oxide,  *NF-Kβ* nuclear factor kappa-light-chain-enhancer of activated β-cells, *MPO* myeloperoxidase, *Gly* glycine, *Pro* proline & *OH-Pro* hydroxyproline. F1: hydrogel containing petroleum ether fraction, F2: hydrogel containing chitosan loaded petroleum ether nanoparticles, F3: hydrogel containing ethyl acetate fraction, F4: hydrogel containing chitosan loaded ethyl acetate nanoparticles

##### Histopathological study

**Fig. 3:**
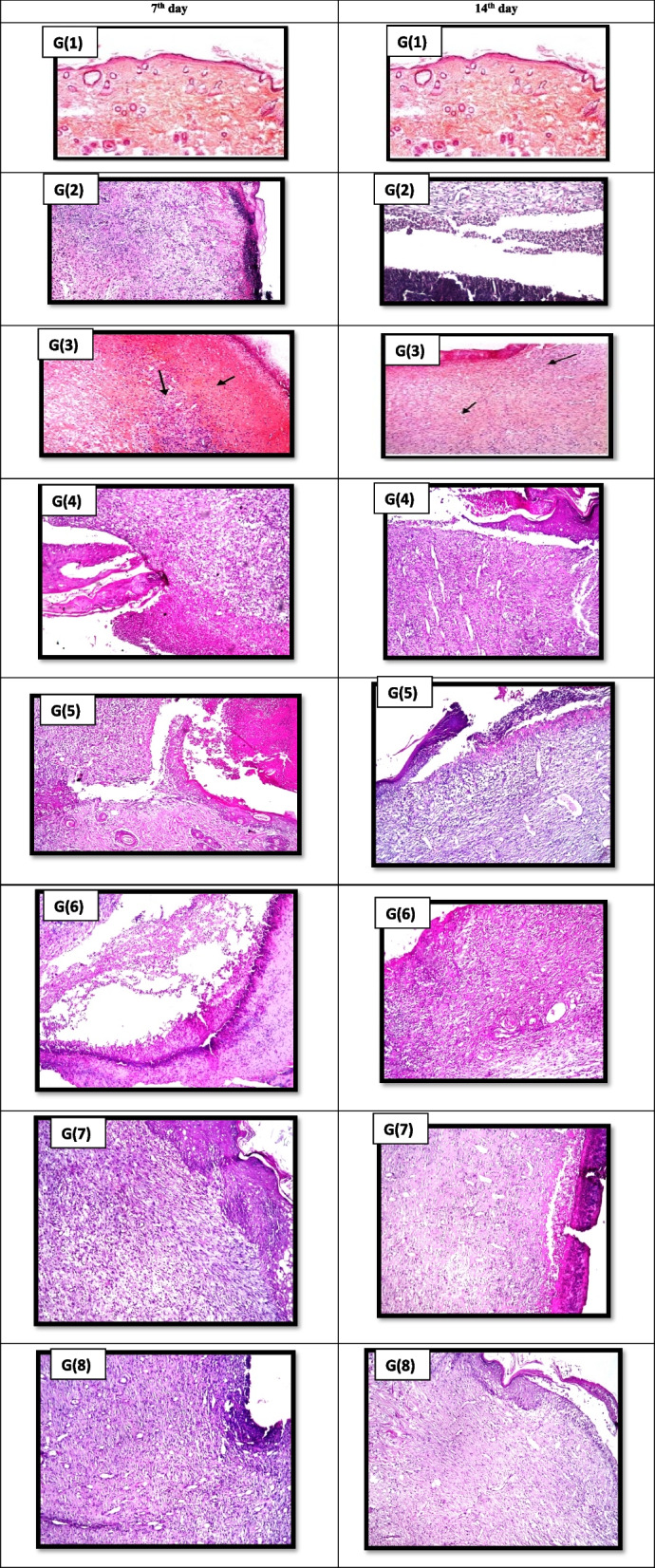
Photomicrographs of normal skin rats (G1) and wounded skin (stained with H&E) in different groups after 7 & 14 days of circular excision wound model. G2: Untreated group, G3 – G8 Groups treated with vehicle (gel base), Panthenol, formulations F1-F4, respectively. St: standard drug (Panthenol gel)

Figure 3 revealed that F4 showed the best result regarding granulation tissue formation that became massive in the 14th day and acanthosis with absence of inflammation in both days which is better than that obtained by Panthenol gel where inflammation and ulceration remain till the 14th day followed by those treated with F1that showed some inflammation in the 14th day then F2 and F3 where moderate inflammation with ulceration still present to the 14th day.

### Phytochemical study of the bioactive fractions

#### Chromatographic isolation and identification of the constituents from pet-B fraction

Nine compounds of different groups (P1-P9) were purified; sterols viz. β-sitosterol (P1) and daucosterol (P2), sesquiterpene quinones as mansonone C (P3) & mansonone E (P4), terpenes as lupeol (P5), betulin (P6), lupeol acetate (P7), alcohol as myricyl alcohol (P8) and acid as 23-hydroxybetulinic acid (P9). Their structures were elucidated by melting points, spectral data (UV, IR, ^1^H-NMR &^13^C-NMR) and comparing the obtained data with those in the published literature.

#### Chromatographic isolation and identification of the constituents from Etac-B fraction

Fifteen compounds (E1-E15) were isolated and identified; Anthocyanins as Cyanidin-3-rutinoside (E1) and Cyanidin-3-sambubioside (E2), Cyanidin-3-(2-glucosyl) glucoside (E3), Delphinidin 3-sambubioside (E4) and flavonoids like 3, 5, 7-Trihydroxy-4H-chromen-4-one (E5), quercetin-3-O-β-D-glucoside (E6), Gossypetin 3-O-glucoside (E7), Kaempferol-3-O-α-D-rhamnoside (E8), vitexin (E9), catechin (E10), rutin (E11) and Kaempferol-3-O-neohesperidoside (E12). Isoflavonoids like Genistin (E13), Daidzin (E14) and Formononetin 7-O-glucoside (E15). Their structures were elucidated by melting points, spectral data (UV, IR, ^1^H-NMR &^13^C-NMR) and comparing the obtained data with those in the published literature.

E1, E2 & E3 were isolated for the first time from *Thespesia populnea* (L.) and were major compounds in the ethyl acetate fraction.

### Quantitative estimation of main active constituents in the bioactive fractions

The results of β-sitosterol content in Table 6 revealed that Pet-B contained 17.72% of β-sitosterol, where the highest percentage was observed in the F1, followed by Pet-B-CLN and finally F2. While the flavonoid content was found to be 17% expressed as rutin, where the highest percentage was observed in F4, followed by F3 and finally Etac-B-CLN.

**Table 6:** Percentages of total sterols as β- sitosterol and total flavonoids as rutin in the bioactive fractions and their hydrogel formulations

Tested substance	Total sterols as β-sitosterol (%)	Total flavonoids as rutin (%)
Pet-B	17.72 ± 1.2	–
Pet-B-CLN	15.08 ± 0.9	–
F1	17.1 ± 1.1	–
F2	13.31 ± 0.8	–
Etac-B	–	17 ± 1.2
Etac-B-CLN	–	15.73 ± 0.6
F3	–	16 ± 0.7
F4	–	16.18 ± 0.5

*Pet-B* Petroleum ether fraction, *Pet-B-CLN* Petroleum ether fraction encapsulated in chitosan nanoparticles, *F*1 Hydrogel containing Pet-B fraction, *F*2 Hydrogel containing Pet-B-CLN, *Etac-B* Ethyl acetate fraction, *Etac-B-CLN* Etac fraction encapsulated in chitosan nanoparticles, *F*3 Hydrogel containing Etac-B fraction, *F*4 Hydrogel containing chitosan Etac-B-CLN

## Discussion

Wound healing is a complex natural process, which is characterized by tissue regeneration. The purpose of wound management is always to enhance the healing process while minimizing risk factors like infection, which have a substantial effect on it. Studies in this area are conducted using a variety of medicinal plants with wound-healing characteristics [45]. The present study evaluated *Thespesia populnea* (L.) bark ethanolic extract and its successive fractions (1 & 2%) on wound healing activity to determine the most bioactive fraction and the most appropriate dose. In addition, the wound healing activity of the prepared hydrogel formulations either through the direct incorporation of the most bioactive fractions into hydrogel formulations or loaded into chitosan nanoparticles were evaluated. The results demonstrated that Pet-B and Etac-B fractions showed the highest wound healing activity at dose 2%. The biological study of the prepared hydrogel formulations of the bioactive fractions revealed that the activity of Pet-B when incorporated in conventional hydrogel is better than that obtained from the hydrogel containing Pet-B-CLN, otherwise, hydrogel formulation containing Etac-B-CLN showed more wound healing activity than that obtained from the conventional hydrogel, supporting the importance of nano-formulations in improving the wound healing activity. Fathima Naslath and Varnakulendran, 2017 reported that stem bark powder of *Thespesia populnea* (L.) showed significant wound healing activity, which is inconsistent with our study confirming its potential wound healing activity [18].

Chitosan is a chitin derivative and biopolymer extracted from the shellfish exoskeleton. Chitosan was loaded with many plant materials and possess high permeability through biological membranes, higher encapsulation performance, being non-toxic and inexpensive [46]. It acts as both drug vehicle together with its wound healing activity through different routes of action as anti-oxidant, anti-inflammatory and anti-microbial activities [47]. Chitosan acts a penetration enhancer by opening the tight junctions of the epithelium and facilitates both paracellular and transcellular transport of drugs [48], when it is used as polymer for nanoparticles formation it proved to increase dug stability and its accumulation as reported by Manne et al., 2021 [49]. Thus, chitosan was selected in this study. Nanoparticles applications are increasing nowadays especially in drug delivery systems. One of the aims of this study is the preparation of nanoparticles to increase and accelerate the efficacy of wound healing activity through enhanced permeation and retention effect with the targeted tissue. Since the plant extract together with the chitosan-loaded nanoparticles are in the powdered form, the topical hydrogel formulations were prepared to perform a more convenient application on the skin, also they increase the residence time of the active constituents to the target tissue and decrease the loss of the active constituent during application [50].

Evaluation of nanoparticles revealed that Pet-B-CLN showed less particle size and more convenient polydispersity index (0.69) that indicates that the samples have a very broad size distribution. The zeta potential of both fractions nanoparticles are close to each other and within the range of − 30 to + 30 indicating large repulsive forces between particles that can prevent aggregation and ensure easy dispersion, indicating more stability in the suspension, where zeta potential is an index of nanoparticles stability, the higher the zeta potential, the more stable suspension is produced [51]. Etac-B-CLN showed better results concerning entrapment efficacy and loading capacity than that of Pet-B-CLN, that may be due to the hydroxyl (OH) groups located in flavonoids that increase their anionic property thus interacting with chitosan either by ionic or hydrogen bond or both leading to complete trapping inside the polymeric matrix of chitosan nanoparticles, while petroleum ether fraction that is rich in sterols, triterpenes and hydrocarbons, is mainly neutral, that reduced the interaction with chitosan so, lower entrapment efficacy and loading capacity were accomplished.

The study of spreadability and rheological properties of the selected hydrogel formulations F1 & F4 revealed the satisfactory results of spreading extent of the formulations on the application surface upon applying low shear and the absence of noticeable effect of the fraction type either normal-sized or chitosan loaded nanoparticles on the rheological behavior and spreadability of the prepared hydrogel formulations. The curves that graphically represented shear stress vs shear rate showed low hysteresis loops, while «ascending» curve, which describes the hydrogel destruction, differ from the «descending» curve, which indicates the resumption of the hydrogel, this is explained by preservation of residual deformation after a strong attenuation of the structure under the influence of earlier applied stress. This rheological behavior characterizes satisfactory spreadability on the skin and the ability to extrusion from the tube [43].

Formulation (F4) shows better results concerning wound healing activity than that obtained from F2, although both contains chitosan loaded nano particles and F2 shows lower particle size than F4, that could be attributed to the loading capacity and entrapment efficacy factors, leading to better absorption and efficacy in F4. The success of Etac-B-CLN to achieve better results than conventional hydrogel formulation F3 could be as a result of its smaller particle size aiding the absorption of the extract through biological membrane. While in case of Pet-B incorporated in conventional hydrogel formulation F1 showed better results than that of chitosan loaded nanoparticles F2 due to less entrapment of its active ingredients especially β-sitosterol with chitosan, resulting in somewhat less wound healing activity than that obtained from the conventional hydrogel formulation (Chitosan loaded nanoparticles did not improve the wound healing activity of Pet-B).

ROS are considered to be crucial factor in delaying the healing process, the anti-oxidant activity was assessed by measuring the level of GSH and MDA that gives an indication to the level of lipid peroxidation [52]. NO has been implicated in both inflammation and wound healing through regulation of epithelization and vasodilatation [53]. Most wounds heal via formation of scar tissue which is mainly composed of collagen. Synthesis of collagen is the most critical step in the process of wound healing that require amino acid precursors viz. glycine, proline and hydroxyproline [4]. Deposition of collagen is maintained during the granulation tissue formation which is converted into complex structure during maturation phase. NF-Kβ that increased during inflammation activates the innate immune reaction, proliferation and migration of cells [54]. As neutrophils are normally the first cells recruited at the site of inflammation or injury [55], inflammation was biochemically monitored by measuring the level of MPO which is considered as an indicator for neutrophils accumulation in tissues [56].

The wound healing activity of Pet-B and Etac-B in hydrogel formulations (F1-F4) may be contributed to their anti-oxidant and anti-inflammatory activities exerted through different cascades during the healing process. The anti-oxidant activity exerted by both Pet-B and Etac-B is reflected by elevated GSH and reducing the NO and MDA levels in the 7th and 14th days of the treatment. The anti-oxidant effect could be related to promoting the epithelization and increasing the collagen synthesis which is proved by the increase in the glycine, proline and hydroxyproline (amino acids of the collagen), increase in the viability of collagen fibrils, increasing the collagen fiber strength, improving the blood circulation, preventing cell damage and promoting DNA synthesis [7]. Additionally, the histopathological study illustrated in the 7th day of treatment by F1, led to the formation of granulation tissue indicating the entrance of proliferation phase of wound healing and absence of necrosis as a result of the reduction in the lipid peroxidation and by the 14th day the improvement in healing is accomplished. Meanwhile the histopathological study of F4 showed acanthosis with formation of new epidermal layer and massive granulation tissue in 7th day, as well as in 14th day at which healing was almost completed. Wound area contraction in the 7th day was observed to be higher in case of Etac-B fraction followed by Pet-B fraction, proving that they accelerate the onset of wound closure specially if compared to the positive control or standard Panthenol that was obvious in the histological study by the massive granulation tissue formation and absence of inflammatory infiltrates.

The anti-inflammatory activity of Pet-B and Etac-B was revealed in the healing process through the decrease in the level of MPO and NF-Kβ in both 7th and 14th days of treatment, that resulted in the ending of the inflammatory phase and the entrance of proliferative phase together with the formation of granulation tissue and speeding up the wound closure, [57].

β-sitosterol one of the major constituents of Pet-B was reported to exert its wound healing activity through the inhibition of NF-Kβ via inhibition of COX-2, enhancing the granulation tissue formation and reducing wound healing time [58], increasing glycine, proline, and hydroxyl proline promoting angiogenesis and proliferation leading to increased collagen deposition, together with the reduction in MDA [59]. Lupeol acetate was reported to perform its wound healing activity through the reduction of NF-Kβ, and MPO, reducing iNOS expression and resulting in reduction of NO [60]. Flavonoids and anthocyanins, the main constituents isolated from the Etac-B fraction, were reported to exert wound healing activity through increasing the migration and proliferation of fibroblasts and collagen synthesis. Their anti-oxidant and anti-inflammatory activities led to reducing the ROS and modulating the inflammatory pathways. Flavonoids accelerate proline production in regulation of collagen biosynthesis. This production of proline may be due to glutamine converted to glutamate into the inner cells [61]. Proanthocyanidins prevent enzymatic hydrolysis of collagen that resulted in the increase of the extracellular collagen [62]. Additionally, cyanidin and delphinidin were reported to have synergestic anti-inflammatory effect that aids in the acceleration of wound healing [63].

## Conclusion

The obtained data concluded that the *Thespesia populnea* L. ethanolic bark extract and its bioactive fractions (petroleum ether and ethyl acetate) effectively stimulate wound contraction in excision wound model mainly due to their anti-oxidant activity that promoted the healing and through the stimulation of collagen amino acids production together with the anti-inflammatory effect that helped in exerting strong healing action. The presence of major compounds viz. β-sitosterol and lupeol acetate in the Pet-B fraction and flavonoids together with anthocyanins especially cyanidin and delphinidin in Etac-B may contribute to the wound healing activity of these bioactive fractions. In addition, chitosan encapsulated nanoparticles hydrogel containing the Etac-B fraction showed highly more activity than that exerted by conventional hydrogel containing Etac-B fraction only, proving the importance of nano-formulations in improvement of wound healing. Furthermore, conventional hydrogel formulation was the best designed for Pet-B fraction, where no observed improvement of wound healing due to nanoparticles.

## Data Availability

The datasets used and/or analysed during the current study available from the corresponding author on reasonable request.

## Abbreviations

But-B: Butanol fraction of ethanolic bark extract
EBE: Ethanolic bark extract
Etac-B: Ethyl acetate fraction of ethanolic bark extract
Etac-B-CLN: Ethylacetate fraction loaded in chitosan nanoparticles
Gly: Glycine
GSH: Glutathione
MDA: Malondialdehyde
Met-B: Methylene chloride fraction of bark ethanolic extract
MPO: Myeloperoxidase
NF-Kβ: Nuclear factor kappa-light-chain-enhancer of activated β-cells
NO: Nitric oxide
OH-Pro: Hydroxyproline
Pet-B: Petroleum ether fraction of bark ethanolic extract
Pet-B-CLN: Petroleum ether fraction loaded in chitosan nanoparticles
Pro: Proline
SEM: Standard error of mean
F1: Hydrogel containing petroleum ether fraction
F2: Hydrogel containing chitosan loaded petroleum ether nanoparticles
F3: Hydrogel containing ethyl acetate fraction
F4: Hydrogel containing chitosan loaded ethyl acetate nanoparticles
